# Interaction of MC4R rs17782313 variants and dietary carbohydrate quantity and quality on basal metabolic rate and general and central obesity in overweight/obese women: a cross-sectional study

**DOI:** 10.1186/s12902-022-01023-5

**Published:** 2022-05-10

**Authors:** Shahab Alizadeh, Sara Pooyan, Atieh Mirzababaei, Hana Arghavani, Hossein Hasani, Khadijeh Mirzaei

**Affiliations:** 1 Department of Cellular and Molecular Nutrition, School of Nutritional Sciences and Dietetics, Tehran University of Medical Sciences (TUMS), Tehran, Iran; 2 Department of Community Nutrition, School of Nutritional Sciences and Dietetics, Tehran University of Medical Sciences (TUMS), P.O. Box:14155-6117, Tehran, Iran

**Keywords:** Carbohydrate, Glycemic index, Glycemic load, Basal metabolic rate, Gene–diet interaction, Melanocortin 4 receptor

## Abstract

**Background:**

Recent studies have shown that dietary carbohydrate quantity and quality as well as genetic variants may contribute to determining the metabolic rate and general and central obesity. This study aimed to examine interactions between melanocortin 4 receptor gene (MC4R) rs17782313 and dietary carbohydrate intake, glycemic index (GI), and glycemic load (GL) on body mass index (BMI), waist circumferences (WC), basal metabolic rate (BMR), and BMR/kg in overweight/obese women.

**Methods:**

A total of 282 Iranian women (BMI ≥ 25) aged 18–56 years were enrolled in this cross-sectional study. All participants were assessed for blood parameters, body composition, BMR, and dietary intake. Dietary carbohydrate intake, GI, and GL were determined using a valid, reliable 147-item food frequency questionnaire. MC4R rs17782313 was genotyped by the restriction fragment length polymorphism (PCR-RFLP) method.

**Results:**

After adjustment for age and energy intake, significant interactions were observed between carbohydrate intake and MC4R rs17782313 in terms of BMI (P Interaction = 0.007), WC (P Interaction = 0.02), and BMR/kg (P Interaction = 0.003) in this way that higher carbohydrate intake, compared with lower intake, was associated with an increase in BMI and WC for individuals with C allele carriers (TC + CC genotypes), while related to an increase in BMR/kg for those carrying the TT genotype. No significant interaction was found between MC4R rs17782313 and GI and GL on BMI, WC, BMR/kg, and BMR.

**Conclusions:**

Interactions between the MC4R rs17782313 and carbohydrate intake probably can have an effect on BMI, WC, and BMR/kg in overweight/obese women.

## Background

Obesity has become a leading issue globally [1, 2] that threatens both psychosocial and physical health, contributing to a higher incidence of many chronic complications, such as nonalcoholic fatty liver disease, diabetes, cardiovascular diseases, cancer, psychosocial disorders, and so on [3–6]. Unlike general obesity, defined as elevated total body adiposity irrespective of its distribution, central obesity represent a high level of abdominal or visceral fat, which is a stronger predictor of obesity-related diseases [7]. Obesity is a multifactorial phenomenon, resulting from an interaction between genetic variants and environmental factors, including diet, physical inactivity, low sleep quality, obesogens, and even the diversity and composition of gut microbiota [8, 9]. Also, a low basal metabolic rate (BMR), as a leading component of total daily energy expenditure, is one of the main metabolic predictors for the development of body weight gain [10].

Recently, the contribution of carbohydrate quality and quantity as measured by the glycemic index (GI) and glycemic load (GL) to obesity has received remarkable attention [11]. Diets with a high GI or GL are quickly digested, absorbed, and raise blood glucose more quickly, which this process accelerates the fluctuations of insulin and glucose, leading to the early return of hunger and excessive calorie intake [12]. In contrast, low GI and GL diets, because of the slower release of glucose and insulin after consumption, stimulate prolonged satiety, decrease food consumption, reduce fat storage by increasing fat oxidation and decreasing lipogenesis [13], limit the reduction in BMR in the fasting condition [14], and in turn may reduce the risk of obesity [15]. Nevertheless, the relation of the quality and quantity of carbohydrate on obesity is currently unknown. Studies investigating the association between measures of obesity and carbohydrates, GI or GL in the diet so far have reported inconsistent results, with positive, null, or negative relationships [13, 16–20]. The diversity of these associations is possibly due to variations in genetic background and gene-diet interactions [21, 22]. Mutations in the Melanocortin-4-receptor (MC4R) gene is the most prevalent monogenic cause of obesity [23], with a prevalence of 1.7–3.0% among obese people [24]. In this sense, variants in the rs17782313 near the MC4R have been strongly related to obesity [25] and showed a significant association with the dietary intake [26], total energy intake [27], increased snacking, as well as hunger [28]. The quality and quantity of dietary carbohydrates affect serum levels of insulin [29]. It is well recognized that brain insulin has a specific role in feeding behavior and satiety [30] and genetic variations in an obesity risk MC4R rs17782313 affect cerebrocortical insulin signaling, resulting in an impaired insulin response in the human brain [31], indicating a possible interaction. It remains, however, to explore the effect of MC4R rs17782313 polymorphism on the association of dietary carbohydrate quantity and quality with obesity and energy expenditure. Although, there is no evidence related to gender impacts on the MC4R gene, gender affects the metabolic rate [32]. A low metabolic rate has been suggested as a likely cause for the elevated adiposity commonly observed in women compared with men [33]. Thus, to precisely investigate and remove gender-specific associations, the present study was undertaken to assess the interactions of MC4R rs17782313 and dietary carbohydrate, GI, and GL on BMR and general and central obesity in overweight/obese women.

## Methods

### Study population

A total of 282 overweight/obese women aged18-56 (mean: 36.49 ± 8.38) years with BMI 25.2–49.60 (mean: 31.04 ± 4.31) were recruited for the present cross-sectional study. The study was performed from October 2014 to January 2015 from women who attended health centers in Tehran, Iran. The inclusion criteria were: body mass index (BMI) 25 or more, age ≥ 18, absence of any acute or chronic infection, no drug/ alcohol abuse, and not being lactating or pregnant at the time of the study. Subjects with an inflammatory disease or with a history of diabetes, thyroid disease, cardiovascular disease, stroke, cancer, liver or kidney disease, and sustained hypertension were excluded because of possible disease-associated alterations in their diet. We also excluded subjects taking vitamin or mineral supplements, hormone therapy, herbal medicines, and corticosteroids, and participants with total daily energy intake ˂800 kcal/ day or ˃ 4200 kcal/day based on the food frequency questionnaire (FFQ). The study protocol has approved by the ethics committee of Endocrinology and Metabolism Research Center of Tehran University of Medical Sciences (TUMS) (ID: IR.TUMS.VCR. REC 97-03-161-41155) and written informed consent was obtained from all participants.

### Sample size calculation

The sample size was calculated based on the method of Peduzzi et al. [34] using the following formula, in which, p is the proportion of cases in the population and k the number of independent variables, then the minimum number of cases to include is: *N* = 10 k / p. Considering that we had 4 independent variables to include in the model and the proportion of obesity in the Iranian population was 0.22 [35], the minimum number of participants required is: *N* = 10 × 4 / 0.22 = 182. Because of the availability of participants in the health centers, we included a total of 282 subjects in the study.

### BMR, body composition, physical activity, and anthropometric measurements

The body composition of all subjects was measured using a Body Composition Analyzer (InBody770 scanner; InBody, Seoul, Korea) according to the manufacturer’s protocol as reported previously in detail [2]. BMR was measured by an expert nutritionist with the use of a Fitmate TM calorimeter (Cosmed Company, Rome, Italy). After 10–12 h overnight fasting and resting, the BMR was registered with the person lying awake and completely at rest. Physical activity was evaluated using a reliable and validated International Physical Activity Questionnaire (IPAQ) [36]. Height and weight were measured in participants with shoes off in standing position and light clothing to the nearest 0.5 cm and 0.1 kg, respectively. BMI was then calculated as body weight (kg) divided by height squared (m2). Waist circumference was assessed at a point midway between the iliac crest and lower rib margin with the use of a Seca tape to the nearest 0.5 cm. The hip circumference of subjects was also measured in the largest part of the hip. Blood pressure (BP) of all subjects was assessed following a 15- min rest by a standard calibrated mercury sphygmomanometer on the right arm. All assessments were carried out by one trained nutritionist to prevent the individual error.

### Dietary assessment

The usual dietary intake of participants during the past year was assessed using a 147-items of FFQ, which its validity and reliability have already been approved [37]. Portion sizes of consumed food items were reported in household measures and were then converted to grams of intake. Nutritionist 4 software was applied to compute nutrient and energy contents of consumed foods. The GI for carbohydrate-containing food items (foods with ≥ 5 g carbohydrate per 100 g or 100 mL) was calculated with the use of average GI values from the GI table reported by Foster-Powell et al. [38], considering glucose as the reference food (GI = 100). The average daily dietary GI was calculated by multiplying the GI of individual foods by the percentage of total energy contributed by carbohydrate {$\sum$ [GI of food item * (grams carbohydrate of consumed food/ total grams of carbohydrate consumed per day)]}. Dietary GL was calculated as [(daily GI * grams carbohydrate consumed per day) /100].

#### Laboratory assays

Serum samples (5 mL) were obtained following 10 to12 hours of overnight period. Fasting blood sugar (FBS) was measured using the glucose oxidase phenol 4-Aminoantipyrine Peroxidase (GOD/PAP) method. Serum level of high-sensitive C-reactive protein (hs-CRP) was assessed by immunoturbidimetric assay. Lipid profile of participants was assessed using enzymatic methods. All measurements were done by an automatic analysis system (Autoanalyzer; Hitachi Ltd, Tokyo, Japan) with Randox laboratories kits.

#### DNA extraction and determination of MC4R rs17782313 variants

 The extraction of genomic DNA from blood samples was performed with the use of the GeneALL DNA extraction kit (Type G Exgene; Genall; Korea) based on the manufacturer’s protocol. The concentration and purity of the extracted DNA were assessed using the Nano Drop spectrophotometer (Thermo Scientific Company, USA). The extracted DNA was stored at 4 C until sequencing was performed. The MC4R rs17782313 single nucleotide polymorphism (SNP) (major allele: T; minor allele: C) was genotyped by polymerase chain reaction-restricted length polymorphism (PCR–RFLP) technique using forward (5-AAGTTCTACCTACCATGTTCTTGG-3) and reverse (5-TTCCCCCTGAAGCTTTTCTTGTCATTTTGAT-3) primers. PCR reactions were performed in a final volume of 20 µl contains 50 ng/µl extracted DNA, 10 pmol/µl from each primer (0.5 µl), 10 µl Permix (Amplicon, Germany), and 8 µl Distilled water with the following conditions in a DNA thermocycler: primary denaturation at 95 °C for 2 min; 35 cycles of denaturation at 95 °C for 30 s, annealing at 58 °C for 30 s, extension at 72 °C for 30 s; with a final extension at 72 °C for 5 min; 4- final step at 4 °C. Amplified DNA (7 µl) was digested with 0.5 µl of BCII restriction enzyme at 56 °C overnight. All products were visualized by electrophoresis in agarose gel. Fragments containing three likely genotypes were then distinguished: CC, CT, and TT.

### Statistical analyses

MC4R genotypes were recoded based on risk allele: code 0 for TT and 1 for TC + CC. Carbohydrate intake, GI, and GL were stratified into low and high categories based on the medians (category 1: lower than the median, category 2: higher than the median), in which, subjects in the category 2 were considered as individuals adhering to a diet with a high GI, GL, or high intake of carbohydrate. Chi-square test was applied to compare qualitative variables across MC4R genotypes (TT vs. TC + CC) and to assess the Hardy–Weinberg equilibrium (HWE). Comparing the quantitative variables between MC4R genotypes was conducted using independent sample test and re-analyzed by analysis of covariance (ANCOVA) to adjust for age, BMI, and physical activity; Notably, for anthropometric and body composition variables, BMI considered as collinear and these variables were adjusted for age and physical activity. Age and energy intake-adjusted generalized linear model (GLM) analysis was used to assess the potential interactions between MC4R genotypes and GI, GL, and carbohydrate intake on BMR, BMR/kg, WC, and BMI. Furthermore, the Pearson correlation coefficient was used to determine the correlation between serum levels of insulin to dietary carbohydrate intake. All analyses were carried out by a statistical Package for Social Science (Version 22.0; SPSS Inc., Chicago IL, USA). *P* < 0.05 was considered statistically significant.

## Results

Genotype and allele frequencies of MC4R rs17782313 SNP among 282 Iranian women who participated in this study are reported in Table 1. The prevalence of the T allele was 54.04%, while the C allele was 45.96%. Participants were categorized based on MC4R rs17782313 genotypes and divided into two groups: TT genotype (*n* = 153) and TC + CC genotype (*n* = 129) (Table 2). Genotypes were not in HWE (*p* ≤ 0.001). All participants were generally (BMI ˃25 kg/m2) and centrally (WC ˃ 80 cm) obese. The mean BMI, WC, BMR, and BMR/kg of the TT genotype carriers were 31.06 ± 4.66 kg/m2, 99.16 ± 10.31 cm, 1391.22 ± 119.50 kcal/day, and 17.35 ± 1.74, respectively, while corresponding values for TC + CC carriers were 30.78 ± 3.90 66 kg/m2, 98.53 ± 9.81 cm, 1368.69 ± 120.51 kcal/day, and 17.29 ± 1.50, respectively, with no significant difference between two groups. There was no significant difference between genotypes in terms of demography, anthropometry, body composition, and insulin and other blood parameters, as well as physical activity, marital status, smoking status, and education level. After adjustment for covariates including age and physical activity, a significant difference in height (*p* = 0.04) was observed between the two groups (Table 2). After adjustment for calorie intake, no significant difference in food intake was revealed between the MC4R rs17782313 genotypes (Table 3). Moreover, no significant correlation was found between serum insulin levels and dietary carbohydrate intake (*r* = − 0.04, *p* = 0.52).

**Table 1:** MC4R rs17782313 genotypes and allelic variants of study population

Alleles frequency		Genotypes frequency		
T	C	TT	TC	CC
*N* = 307 (54.04%)	*N* = 261 (45.96%)	*N *= 120 (42.3%)	*N* = 67 (23.6%)	*N* = 97 (34.1%)

**Table 2:** Characteristics of the study participants according to MC4R rs17782313 genotypes

	Total		MC4R rs17782313 genotypes			
	*N* = 282		TT (*n* = 153)	TC + CC (*n* = 129)	*p*-value	*p*-value*
**Demography and anthropometry**						
Age (year)			37.23 ± 8.65	35.77 ± 8.03	0.14	0.10
Height (cm)			161.90 ± 5.85	160.98 ± 5.81	0.19	0.04a
Weight (kg)			81.06 ± 12.32	80.28 ± 12.48	0.60	0.91a
BMI (kg/m2)			31.06 ± 4.66	30.78 ± 3.90	0.58	0.46a
WC (cm)			99.16 ± 10.31	98.53 ± 9.81	0.60	0.67a
WHR			0.93 ± 0.05	1.64 ± 8.00	0.27	0.49a
**Body composition**						
Fat percentage (%)			41.38 ± 5.56	41.40 ± 5.39	0.97	0.32a
Fat mass (kg)			34.02 ± 8.99	33.68 ± 8.30	0.74	0.42a
FFM (%)			47.27 ± 5.53	46.23 ± 5.57	0.11	0.16a
Visceral fat (%)			16.42 ± 11.41	16.93 ± 17.32	0.76	0.63a
TBW (%)			34.72 ± 4.06	33.98 ± 4.10	0.73	0.19a
Trunk fat (%)			16.55 ± 3.77	16.47 ± 3.64	0.85	0.36a
BMR (kcal/day)			1391.22 ± 119.50	1368.69 ± 120.51	0.11	0.16a
BMR/KG			17.35 ± 1.74	17.29 ± 1.50	0.77	0.17a
**Blood parameters**						
Insulin( IU/mL )			1.24 ± 0.22	1.19 ± 0.24	0.11	0.06
FBS (mg/dL)			87.94 ± 10.21	86.24 ± 8.46	0.16	0.46
TC (mg/dL)			185.19 ± 34.12	184.83 ± 38.08	0.93	0.33
TG (mg/dL)			127.89 ± 74.23	114.52 ± 64.32	0.13	0.31
HDL-C (mg/dL)			46.35 ± 9.94	47.07 ± 11.98	0.60	0.37
LDL-C (mg/dL)			95.81 ± 23.23	93.84 ± 25.24	0.52	0.95
hs-CRP (mg/L)			4.27 ± 4.53	4.62 ± 4.85	0.56	0.17
**Blood pressure**						
SBP (mmHg)			112.16 ± 14.04	110.92 ± 13.78	0.46	0.28
DBP (mmHg)			77.92 ± 9.59	77.49 ± 9.87	0.71	0.50
Physical activity score			1238.64 ± 1892.47	1177.10 ± 2371.62	0.82	0.92
Smoking					0.24	0.20
yes		15	6	9		
no		267	148	119		
Marital status					0.92	0.73
Married		219	120	99		
Single		54	29	25		
Divorced		8	5	3		
Education level					0.11	0.10
Illiterate		3	0	3		
≤ Diploma		119	63	56		
University degree		160	91	69		

Categorical variables are presented as frequency (n), and continuous variables as mean ± SD

*BMI *body mass index, *WC* waist circumference, *WHR *waist-to-hip ratio, *FBS *fasting blood sugar, *FFM *fat free mass, *HDL-C *high density lipoprotein cholesterol, *hs-CRP *high-sensitivity C reactive protein, *LDL-C *low density lipoprotein cholesterol, *BMR *basal metabolic rate, *TBW *Total body water, *TG* triacylglycerol, *TC *total cholesterol, *SBP *systolic blood pressure, *DBP *diastolic blood pressure

* After adjustment for age, BMI and physical activity

^a^ BMI considered as collinear and these variables were adjusted for age and physical activity

**Table 3:** Dietary intake of study population according to MC4R rs17782313 genotypes

	Total	MC4R rs17782313 genotypes			
	*N* = 282	TT (*n* = 153)	TC + CC (*n* = 129)	*p*-value	*p*-value^a^
**Macronutrient**					
Energy (kcal)		2614.27 ± 753.50	2588.40 ± 709.46	0.77	-
Carbohydrate (g)		372.68 ± 123.59	367.74 ± 111.35	0.73	0.84
Protein (g)		89.61 ± 27.72	86.76 ± 29.01	0.40	0.32
Fat (g) )		93.65 ± 31.92	96.04 ± 33.63	0.92	0.59
**Fatty acids**					
Saturated (g)		28.21 ± 11.57	27.34 ± 9.90	0.51	0.52
Polyunsaturated (g)		19.48 ± 8.66	20.41 ± 9.59	0.39	0.24
Monounsaturated (g)		30.89 ± 11.17	31.37 ± 12.38	0.73	0.48
Linoleic (g)		16.77 ± 8.22	17.67 ± 9.06	0.38	0.26
Linolenic (g)		1.21 ± 0.66	1.25 ± 0.70	0.56	0.41
Oleic (g)		27.67 ± 10.70	28.21 ± 11.84	0.69	0.47
Trans (g)		0.009 ± 0.002	0.009 ± 0.002	0.85	0.86
**Minerals**					
Selenium (mg)		122.61 ± 45.65	115.40 ± 38.36	0.16	0.08
Calcium (mg)		1180.02 ± 421.32	1126.84 ± 394.45	0.28	0.23
Phosphorus (mg)		1657.13 ± 520.98	1594.45 ± 495.01	0.31	0.18
Magnesium (mg)		467.57 ± 153.76	443.32 ± 135.56	0.17	0.06
Zinc (mg)		13.00 ± 4.13	12.67 ± 4.11	0.51	0.45
Iron (mg)		18.67 ± 5.92	18.38 ± 5.84	0.67	0.74
Copper (mg)		1.98 ± 0.65	1.97 ± 0.77	0.90	0.86
**Vitamins**					
E (mg)		16.92 ± 8.51	17.44 ± 9.63	0.63	0.54
D (µg)		1.94 ± 1.67	1.94 ± 1.47	0.99	0.91
A (RAE)		802.96 ± 438.62	734.13 ± 351.96	0.15	0.15
K (µg)		229.11 ± 212.74	197.71 ± 170.86	0.18	0.19
B1 (mg)		2.10 ± 0.67	2.03 ± 0.62	0.33	0.18
B2 (mg)		2.19 ± 0.75	2.16 ± 0.84	0.72	0.83
B3 (mg)		25.30 ± 8.42	25.02 ± 10.06	0.80	0.96
B5 (mg)		6.51 ± 2.10	6.42 ± 2.73	0.76	0.88
B6 (mg)		2.20 ± 0.69	2.12 ± 0.72	0.33	0.23
B9 (µg)		614.13 ± 179.57	591.83 ± 169.77	0.73	0.17
B12 (µg)		4.31 ± 2.21	4.30 ± 2.25	0.97	0.90
C (mg)		196.48 ± 114.00	190.16 ± 138.70	0.67	0.75
**Others**					
Caffeine (mg)		159.39 ± 184.84	138.71 ± 98.94	0.26	0.28
Fiber (g)		45.07 ± 19.28	44.31 ± 17.59	0.73	0.84

^a^After adjustment for calories intake

**Fig. 1:**
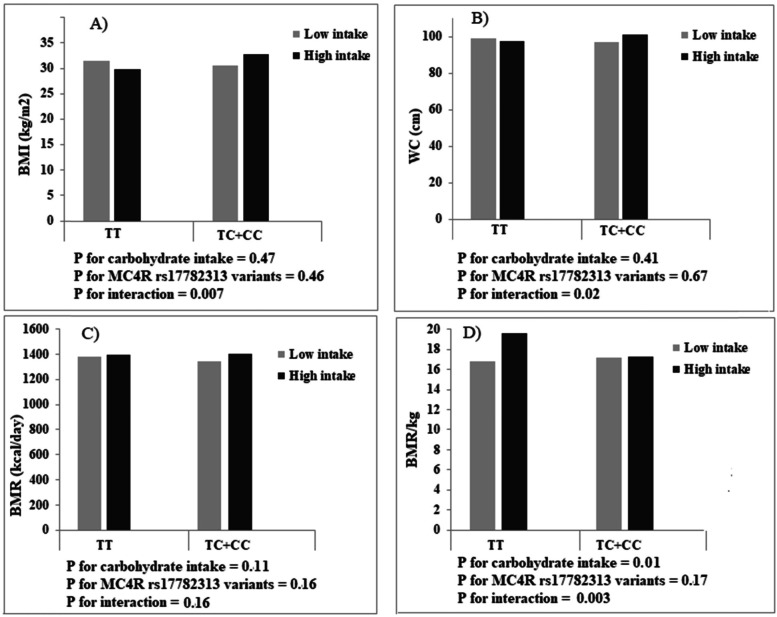
Interactions between carbohydrate intake and MC4R rs17782313 genotypes on body mass index (BMI), waist circumference (WC), basal metabolic rate (BMR), and basal metabolic rate/kg (BMR/kg)

**Fig. 2:**
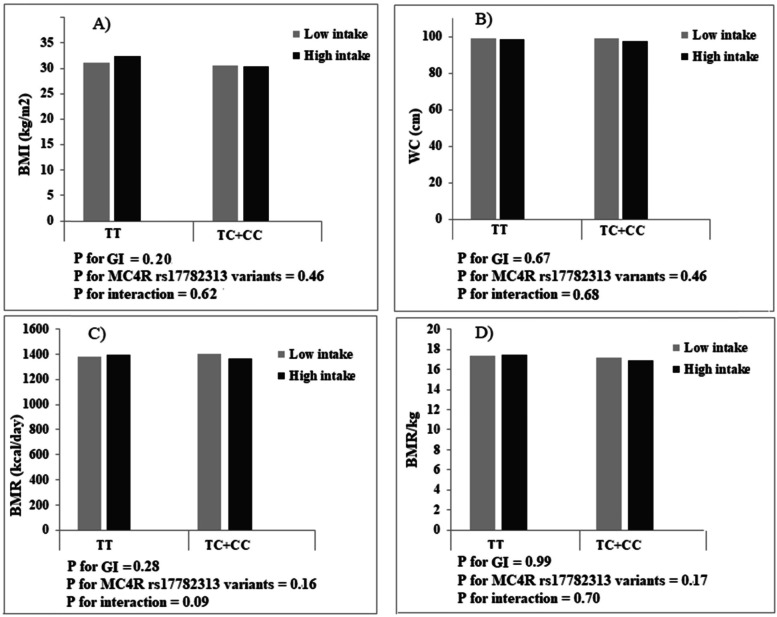
Interactions between glycemic index and MC4R rs17782313 genotypes on body mass index (BMI),), waist circumference (WC), basal metabolic rate (BMR), and basal metabolic rate/kg (BMR/kg)

**Fig. 3:**
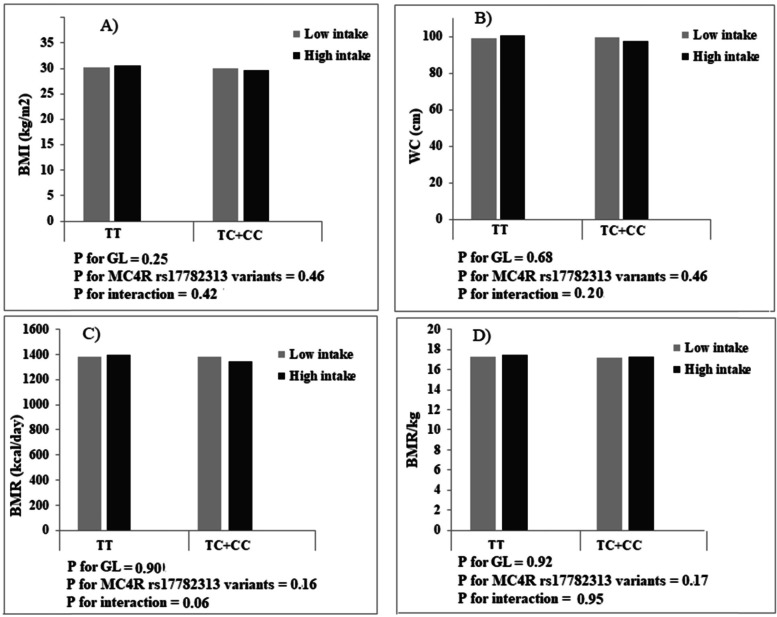
Interactions between glycemic load and MC4R rs17782313 genotypes on body mass index (BMI),), waist circumference (WC), basal metabolic rate (BMR), and basal metabolic rate/kg (BMR/kg)

Comparing women who consumed a diet with GI, GL, or carbohydrate less than the median with women who consumed higher than the median, there were no differences in BMI, WC, and BMR (*p* > 0.05), but women with higher carbohydrate intake, compared with those with lower intake, had a height BMR/kg (low intake: 17.16 ± 1.53 vs. high intake: 17.41 ± 1.74; *p* = 0.01). After adjustment for age and energy intake, significant interactions were observed between dietary carbohydrate intake and MC4R rs17782313 in terms of BMI (P Interaction = 0.007), WC (P Interaction = 0.02), and BMR/kg (P Interaction = 0.003) in this way that higher carbohydrate intake, compared with lower intake, was associated with an increase in BMI and WC for individuals with C allele carriers (TC + CC genotypes), while related to an increase in BMR/kg for those carrying the TT genotype (Fig. 1). No significant interaction was found between MC4R rs17782313 and GI (Fig. 2) and GL (Fig. 3) on BMI, WC, BMR/kg, and BMR.

## Discussion

Dietary intake is one of the leading contributing factors to obesity; besides, genetic variants potentially affect daily energy expenditure and obesity. In an attempt to recognize factors involved in susceptibility to being overweight/obese, this study aimed to examine the interaction between dietary carbohydrate intake, GI, and GL and MC4R rs17782313 on BMR and measures of central and general obesity in overweight/obese women. The main finding of this study is that MC4R SNP can modify the association between dietary carbohydrate intake and BMR, BMI, and WC. Women with high carbohydrate intake and the TT genotype of MC4R rs17782313 had a higher BMR/kg; moreover, higher carbohydrate intake was associated with an increase in BMI and WC in individuals with risk allele (TC + CC genotypes).

For *MC4R* rs17782313, most studies, in agreement with or findings, found no significant association between this SNP and dietary carbohydrate and energy intake [39, 40]. Although, limited evidence revealed that carriers of the CC genotype may have a higher energy intake and a lower carbohydrate intake, compared with individuals with the TT genotype [27]. Our results regarding higher BMR/kg in persons consuming a high carbohydrate diet and TT genotype of *MC4R* is supported by previous studies showing that energy expenditure is higher after carbohydrate overfeeding [41]. The metabolic rate is decreased after adherence to a low-carbohydrate diet via mechanisms associated with substrate availability, and autonomic and hormonal activity [14, 42]. Clinical trial studies also identified that the reduced effectiveness of low-carbohydrate diets over time is because of metabolic depression [43]. Tentolouris et al. [44] and Potter et al. [45] showed that higher energy expenditure after a high-carbohydrate meal is due to increased activity of the sympathetic nervous system, which is not seen following a high-fat meal.

The relation of carbohydrate amount and quality in promoting obesity has been debating, with inconclusive epidemiologic results for the contribution of dietary carbohydrate intake, GL, and GI to long-term weight gain [13, 16–20]. Earlier reports from Asian [46] and Mediterranean [47] populations showed that carbohydrate-rich diets are not positively related to any measure of obesity. In contrast, in another study, higher dietary total carbohydrate and GL were related to a diminished probability of being obese (BMI ≥ 30 kg/m2), while GI did not relate to obesity [48]. In agreement with our findings, a cross-sectional study from the Iranian population identified no significant relationship between dietary GI and abdominal as well as general obesity; however, an inverse link was reported between GL and BMI and WC [49]. Another cross-sectional study on 979 adults, in line with our study, reported no association between GI and GL with either WC or BMI [17]. In contrast, some studies from the US [16] and Japan [50] reported a positive link between GI and BMI. In spite of this, a negative relationship between dietary GI or GL and measures of obesity was reported in some other investigations [18, 51]. Based on our findings, it is probable that, at least in the present population, only the quantity of carbohydrate is determinant for the prediction of obesity rather than quality. One of the reasons for the lack of association between GI and measures of obesity in the present study could be that high-carbohydrate diets may override any impacts of GI [52]. Moreover, heterogeneity in the findings may result from the discrepancy in the subject’s characteristics, dietary assessment methods used, study design, study sample size, and lack of controlling for covariates. Further studies are needed to investigate this area of research.

Our study revealed that the relation of carbohydrate intake to BMR and measures of obesity depends on MC4R polymorphism. The MC4R is a fundamental regulator of energy balance via functionally divergent central melanocortin neuronal pathways, affecting energy expenditure and food intake [53]. Rs17782313 is shown to be significantly related to visceral fat accumulation, WC, and BMI so that each additional C allele of rs17782313 displayed an elevated weight by 2.45 kg [54]. Animal evidence has shown that mice with MC4R knockout mutations have elevated food intake and decreased energy expenditure [55]. Mice lacking MC4R also have lower energy expenditure in comparison with leptin knockout mice [56]. MC4R knockout mice progress obesity possibly as a result of reduced energy expenditure even in case of stable food intake [57]. This shows that the *MC4R* gene could control both energy intake and energy expenditure, supporting the evidence that, in rodents, MC4R contributes to mediating energy homeostasis, food intake, and obesity. Though evidence on the relation of MC4R SNP to energy expenditure is partial in humans, we found that its mutated variants are associated with indices of obesity and decreased energy expenditure by having an interaction with dietary carbohydrate intake. Iranians consume a large amount of carbohydrate in their usual diets [49], which in case of being the carrier of MC4R rs17782313 risk allele, may be at higher risk for general and central obesity. Dietary carbohydrate is the most potent inducer of insulin release, which substantially stimulates the synthesis, uptake, and storage of fat in adipose tissue [46]; this underlies the hypothesis that high-carbohydrate diets induce accumulation of excess adiposity and obesity. Modulation of the relation of carbohydrate to WC and BMI by MC4R SNP may partly justify the inconsistent results of previous studies investigating the association of carbohydrate intake with obesity risk. The potential clinical importance of gene-diet interaction observed in our study is to develop personalized dietary approaches for the prevention of obesity and its related metabolic disorders according to the genetic background that is modified to meet individual’s requirements.

Insulin is a neuromodulator of energy homeostasis, a marker of energy stores, a mediator of energy balance, and an adiposity signal [30]. Hypothalamic or intra-cerebroventricular delivery of insulin, as a leptogenic and anorexigenic signal, decreases food intake, and yields a continuing weight loss in both primates [58] and rodents [59, 60]. Also, neuron-specific insulin receptor “knockout” mice display an elevation in food intake and body weight [61]. Extracellular concentrations of insulin in the hypothalamus are increased in response to a carbohydrate meal [30]. On the other side, it has been shown that insulin response in the human brain is impaired in C allele carriers (TC/CC) of the *MC4R* rs17782313 [31]. Therefore, mechanistically, higher carbohydrate intake contributes to an increase in obesity indices in individuals with the C allele (TC + CC genotypes), because brain insulin concentration is elevated in response to carbohydrate [30], while the C allele of *MC4R* rs17782313 is associated with cerebrocortical insulin resistance [31], resulting in a reduction in its anorexigenic effects, which ultimately their interactive effect leads to the increased body weight. These justify the interaction observed between MC4R rs17782313 variants and dietary carbohydrate intake on indices of obesity. As strength points, to the best of our knowledge, this is the first study to identify an interaction between dietary carbohydrate and MC4R rs17782313 for the susceptibility to increased measures of central and general obesity as well as metabolic rate. Also, several measurable covariates such as energy intake, age, and physical activity were measured and adjusted for. Some limitations of this study should be considered. First, because of the cross-sectional nature of the present study, causality cannot be inferred; moreover, it was not possible to determine the mechanism of the relationship between carbohydrate intake and the rs17782313 genotype. Thus, further investigations, particularly clinical trials or prospective studies, are required to confirm our results. Second, this study included a relatively small number of subjects and restricted participants to women, which limits the generalizability of findings. Accordingly, it is proposed to conduct similar investigations with a larger sample size on both genders. Third, we assessed dietary intake using a validated FFQ; though, FFQ is prone to measurement error and recall bias. Forth, genotype frequencies did not follow the HWE. Our study is a part of a larger study, in which data regarding MC4R rs17782313 variants was available in 282 subjects; we included these participants, and deviation from HWE might result from this reason that the study participants are a part of a larger study. Deviation from the Hardy-Weinberg equilibrium has been reported in many gene-disease association studies [62–64]. Nevertheless, this deviation may limit the generalizability of the findings. Finally, in the present study, only the most common SNP in the MC4R gene was examined and other SNPs are suggested to be investigated by future similar studies.

## Conclusions

In conclusion, this study for the first time provided preliminary evidence that there may be an interactive effect between carbohydrate intake and the MC4R rs17782313 genotypes on BMR, BMI, and WC. We found that adherence to a high-carbohydrate diet in subjects with the TT genotype of MC4R rs17782313 is related to a higher BMR/kg. These data also emphasize that individuals with the C allele of rs17782313 in MC4R following a high-carbohydrate diet had higher BMI and WC; indeed, these people are more susceptible to being overweight/obese with high- carbohydrate diets. Additional studies are required by including the clinical level to clarify the biology of MC4R rs17782313 and its impact on the relationship between dietary carbohydrate intake, metabolic rate, and degree of obesity.

## Data Availability

The Data are not publicly available because of containing information that could compromise the privacy of research.

## Abbreviations

MC4R: melanocortin 4 receptor
GI: glycemic index
GL: glycemic load
BMI: body mass index
WC: waist circumferences
BMR: basal metabolic rate
BMR/kg: basal metabolic rate/kilogram
PCR-RFLP: polymerase chain reaction-restriction fragment length polymorphism
FFQ: food frequency questionnaire
IPAQ: international Physical Activity Questionnaire
FBS: Fasting blood sugar
GOD/PAP: glucose oxidase phenol 4-Aminoantipyrine Peroxidase
hs-CRP: high-sensitive C-reactive protein
BP: Blood pressure
SNP: single nucleotide polymorphism
ANCOVA: analysis of covariance
GLM: generalized linear model
WHR: waist-to-hip ratio
FFM: fat free mass
HDL-C: low density lipoprotein cholesterol
TBW: Total body water
TG: triacylglycerol
TC: total cholesterol
SBP: systolic blood pressure
DBP: diastolic blood pressure
